# ‘You Can Get That Person on ART but You Can’t Give Them Back Their Social System’: A Qualitative Analysis of Voluntary Assisted Partner Notification for HIV for Marginalised and Vulnerable Populations

**DOI:** 10.1177/23259582241272059

**Published:** 2024-09-09

**Authors:** Kate Bärnighausen, Astrid Berner-Rodoreda, Maureen McGowan, Mark Donald Reñosa, Caroline Mtaita, Florian Neuhann

**Affiliations:** 1 Faculty of Medicine and University Hospital, Heidelberg Institute of Global Health (HIGH), 199880 Heidelberg University, Heidelberg, Germany; 2 School for Data Science and Computational Thinking, Stellenbosch University, Stellenbosch, South Africa; 3 School of Public Health, 56400 University of the Witwatersrand, Johannesburg, South Africa; 4 Department of Epidemiology and Biostatistics, 433382 Research Institute for Tropical Medicine, Department of Health, Muntinlupa, Philippines; 5 School of Medicine and Clinical Sciences, Levy Mwanawasa Medical University, Lusaka, Zambia

**Keywords:** voluntary assisted partner notification, HIV, marginalised, vulnerable, qualitative

## Abstract

Little is known about Voluntary Assisted Partner Notification (VAPN) in groups in sub-Saharan Africa that experience marginalisation, and whether its use is suitable for referral to HIV care pathways. We conducted semi-structured in-depth interviews with purposively selected medical and health professionals (*N* = 15) regarding their perspectives and experiences with VAPN policy and its implementation. Data were analysed following a Reflexive Thematic Analysis approach. Respondents highlighted the flexibility in VAPN policy implementation and described adjustments made by health workers. Women were seen as vulnerable and lacked access to support against gender-based violence. Men who have sex with men could face exclusion from important social networks. Age-appropriate VAPN assistance was also considered unavailable for sexually active children. Embedding understandings of identity, belonging, and safety into VAPN could address individual priorities and needs. Community support networks, tailored care for children, and family-orientated approaches to HIV notification may overcome issues relating to vulnerability and marginalisation.

## Plain Language Summary

*A qualitative analysis of voluntary assisted partner notification*

Little is known about Voluntary Assisted Partner Notification (VAPN) in groups in sub-Saharan Africa that experience marginalisation. We conducted semi-structured in-depth interviews with purposively selected medical and health professionals (*N* = 15) regarding their perspectives and experiences with VAPN policy and implementation. Data were analysed following a Reflexive Thematic Analysis approach. Respondents highlighted flexibility in VAPN policy implementation and described adjustments made by health workers. Women were seen as vulnerable and lacked access to support against gender-based violence. Men who have sex with men could face exclusion from important social networks. Age-appropriate VAPN assistance was also considered unavailable for sexually active children. Embedding understandings of identity, belonging, and safety into VAPN could address individual priorities and needs. Community support networks, tailored care for children, and family-orientated approaches to HIV notification may overcome issues relating to vulnerability and marginalisation.

## Introduction

With an estimated 650,000 HIV infections annually, the sub-Saharan African (SSA) region requires novel approaches and ongoing commitment to HIV testing, prevention and linking patients to care.^1^ Voluntary Assisted Partner Notification (VAPN) is one established method of identifying HIV infections, with multiple countries adopting this approach as a core strategy within HIV prevention and care programmes.^2^ While much is understood around access, cost and acceptability, little is known about the perceptions of VAPN for marginalised and vulnerable population groups, particularly in the sub-Saharan African context.

VAPN is a public health service which targets partners of persons recently diagnosed with HIV (index clients) and aims to increase HIV prevention and treatment through the identification of new HIV infections, identification of sero-discordant couples, and the provision of voluntary HIV testing services.^2^ VAPN services can be separated into 3 methods of assistance: contract, dual, and provider referral.^3^ Contract referral occurs when the community health worker (CHW) develops a contract with the index client, during which the index client has a specified amount of time (eg, 1-4 weeks) to refer partners to HIV testing services.^4,5^ Should the partner not seek testing during this time period, they are then contacted by the CHW either by phone or through home/work visits.^6^ Dual referral is the process of a healthcare worker accompanying the index client and providing support during disclosure and offering voluntary testing. Last, provider referral occurs when CHW immediately and confidentially contacts partners and explains the risk of exposure while encouraging HIV testing without direct involvement of the index client.^3^

VAPN has long been established to manage the transmission of HIV/AIDS globally^6,7^ and has been attributed to increased rates of new HIV diagnoses by 20% and 21% in the US and UK, respectively.^8,9^ Similarly, pilot studies in Malawi, Tanzania, Cameroon, Kenya, Uganda, and Mozambique have also reported increased rates of VAPN-supported HIV testing, particularly among men.^4^ VAPN supports the identification of sero-discordant couples and – alongside HIV testing services – has been found to be generally acceptable among participants.^10,11^ A study conducted in Malawi (2011) reported a partner testing rate of 42.4% and 62.6% HIV-positive diagnoses among partners. These studies have been referenced as evidence to scale-up VAPN services in the larger sub-Saharan African region.^4^

While VAPN appears to increase testing, partner notification is also associated with increased levels of stigma, fear of social exclusion and health rights violations; mostly related to the pressure applied to individuals to disclose their status to their partners, or patient confidentiality breaches by HCWs.^12^ VAPN is considered to be an effective method of those with one, long-term partnership,^4^ but may require tailoring in high prevalence settings where marginalised individuals have stigmatised partnerships,^13^ and are unable to locate or contact sexual partners.^14^ Here, we use the definition of Hall et al (1994, p. 25) who define social marginalisation as ‘…the process through which persons are peripheralized based on social identities including, but not limited to, gender, class, race, sexual orientation, and socioeconomic status’.^15^ Social marginalisation leads to disparities in health outcomes and intersects with HIV stigma and access to HIV prevention or treatment services.^16^

For vulnerable population groups, such as minors, women at high risk of HIV infection^17^ and marginalised MSM, less is known about interactions with VAPN as a service, and whether it functions as it can for others. As part of the 5 C's approach (consent, confidentiality, counselling, correct results and connections), The World Health Organisation (WHO) and UNAIDS state that testing and notification should be ‘tailored to the person’ and advocate for differentiated VAPN delivery. However, specific to VAPN, there is little guidance as to how to notify minors, or how or if a minor should be involved in the notification process. There is some evidence that vulnerable women and female sex workers (FSW) are reluctant to use VAPN services because of fears relating to intimate partner violence,^18^ stigma, discrimination, and social exclusion.^12^ For MSM, criminalisation of same-sex-relationships^19,20^ is thought to exclude individuals and minimise the ability to access VAPN and other sexual health services.^21,22^

In this article, we examine medical and health professionals’ perceptions of VAPN for marginalised and vulnerable populations in SSA. Our research examined interviewees’ perspectives on VAPN implementation at the community, national and international levels, available VAPN information for implementers, for people living with HIV and the general public, facilitators and barriers to implementation, outcomes in terms of yield, costs, reporting mechanisms and if the practice of VAPN aligns with human rights principles such as rights to the prevention, treatment, and control of disease as well as the rights to confidentiality, anonymity, consent, and remaining free from coercion. Our research seeks to strengthen effective VAPN and appraise the feasibility and acceptability of VAPN for marginalised and vulnerable groups in this diverse region.

## Methods

### Study Setting

This study was conducted online with community, national and international medical and health professionals in Europe, the USA, as well as national and community medical and health professionals across the sub-Saharan African region in Malawi, Kenya, Tanzania, Zimbabwe, Eswatini, Sierra Leone, and South Africa.

### Design

We used an exploratory qualitative design using semi-structured in-depth interviews. Our study methods are reported in accordance with the consolidated criteria for reporting qualitative research (COREQ).^23^

### Sample and Recruitment

We contacted participants that represented the community, national and international levels of VAPN and purposively selected *n* = 30 male and female adults (>18 years) from Malawi, Kenya, Tanzania, Sierra Leone, Zimbabwe, Eswatini, and South Africa. ‘Community level’ refers to those who have worked or are working in the community, and includes participants from non-governmental organisations (NGOs), advocacy and health care. ‘National level’ refers to participants who have worked or are working in the SSA region and includes NGOs, Ministry of Health staff and academics. The ‘international level’ includes participants from academia, multilateral organizations, HIV policy and NGOs working on multiple HIV projects globally. Our purposive criteria included experience in VAPN policy development, and/or VAPN program implementation, and service delivery. Identification of participants was via internet searches of in-country professionals where VAPN was stated as a professional competency or component of their work, and where we were able to conduct the interview in English. Participants were also identified via contacts within our established professional HIV networks. This included networks among policy-makers, those working in NGOs, academics, and health providers. Two-thirds of our participants were selected because they had first-hand delivery experience of VAPN, and the remaining 5 were positioned in policy and implementation roles. We excluded 10 of the original selection because, although HIV professionals, they had limited VAPN experience. The remaining *n* = 20 who agreed to participate met our selection criteria. 2 then did not reply to follow-up requests and 3 could not schedule a time to participate in an interview. *n* = 15 completed the interview. Participants were contacted via email which included the names and roles of the study team and an overview of the study. Those who replied and indicated that they would participate were sent an information sheet and were asked to choose a date and time for the interview.

### Data Collection

From December 2019 to July 2020, we conducted qualitative in-depth interviews using online telecommunications applications. Interviewers (KB and ABR) are experienced female post-doctoral qualitative researchers. Data collection tools designed by the study team included an interview cover sheet to capture participant details, an informed consent sheet, an information sheet and interview guides. The interview guides were piloted within our team and refined to improve question relevance and clarity. Our coversheet also contained a space for reflexive and observational notes. Interview guides were designed to explore perspectives of VAPN reporting, estimated cost, barriers of implementation, facilitators of implementation, perceived outcomes, and perceived alignment with human rights principles. We used targeted guides for the interviewee's scope of intervention (community, national and international). Interviews lasted 45 to 120 min and were audio recorded using a communication application of the participants choosing. We did not conduct follow-up interviews. We conducted debriefings after each interview to refine lines of inquiry, to enable a space for reflexive discussion, to gauge data saturation and to triangulate key findings.^24,25^ Given the sample specificity and the quality of interview dialogue in relation to marginalisation and vulnerability, we felt we had enough ‘information power’ in 15 interviews.^26^

### Data Analysis

Interviews were transcribed in English and 10 were randomly selected to check for data quality and accuracy. Transcripts were not returned to participants. Transcripts, interviewer notes, and debriefings were managed using Nvivo Pro 12 and analysed using the tenets of Reflexive Thematic Analysis (RTA).^27^ This was a recursive process following 6 stages of analysis. KB, ABR and MM independently read and re-read transcripts for data familiarisation. KB and MM inductively analysed transcripts in blocks of five, and together reviewed where codes were similar or diverged. This process was followed until coding was complete. We presented the main codes to the study team and discussed where reoccurring codes could build our core themes. After revising this codebook, KB and MM then independently developed 2 distinct thematic schemes which were presented to the study team, refined, named and finalised for interpretation and writing up. Participants did not provide feedback on the findings.

### Reflexivity Statement

The research team consists of 1 professor, 4 post-doctoral researchers and 1 doctoral researcher. 5 of the team have extensive experience at the academic, multilateral organization, and NGO levels of HIV research, service provision, prevention and care. As this experience is what motivated the study, we considered and challenged our own perceptions frequently in the design of the interview guides, who should conduct the interviews and our interpretation of the data. We shared our thoughts in debriefings and on a bi-weekly basis as a team and recruited 2 external colleagues to review our findings who extensively questioned our reflexive notes and re-framed the ideas and themes we were generating to ensure our personal biases were limited.

### Ethical Approval and Informed Consent

This study received IRB ethical clearance from Heidelberg University Ethics Commission number S-581/2019. As interviews were conducted online, participants received an information sheet via Email or WhatsApp and were asked for the signed consent form to be returned prior to the interview.^28^ Before interviews took place, we ensured that the written informed consent was obtained from all participants. We informed all participants that the interviewer was in a private space, and advised the participant to also choose somewhere that was private and quiet.^28^

## Theoretical Framework

Once our themes were finalised, we began to see a pattern of perceived social risk associated with the HIV status and notification. We felt these patterns built upon and resonated with the aspects of the theory of Social Exchange,^29^ which posits that social behaviour is the result of an exchange process in which the purpose of exchange is to maximise perceived benefits and minimise perceived costs.^29,30^ Social exchange is based on self-interest and interdependence within relationships and the society is viewed as a system in which social interactions consist of a trade-in valued resources.^29^ Here, valued resources include relationships and social networks, safety, health rights, HIV status, and counselling skills. Commitment to actions which are thought to maximise valued resources are influenced by the individual's context, particularly in relation to previous experience within relationships, trust, rewards, and reciprocity.^29,30^ The terms of these commitments are largely influenced by the relative power of those in the exchange and the partner who is least dependent on the exchange for maximising benefits who has the greater bargaining power to improve their outcomes. If the exchange is considered too risky, the relationship within the exchange and or between the 2 parties may be ended.^29,30^

**Figure 1.**
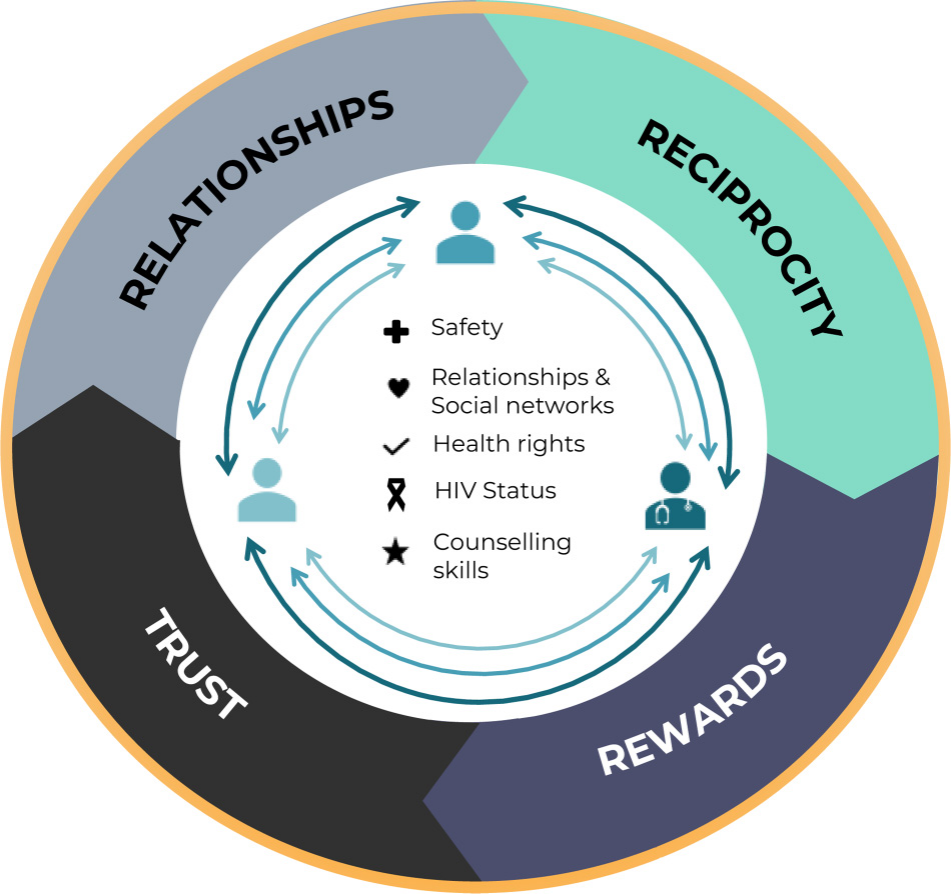
Social exchange framework.

Our adapted model of Social Exchange highlights where – within the exchange – value is placed, and describes the actions taken to maximise benefits and reduce social cost. We include 3 parties in the exchange process; the index client, the partner and the HCW that is assisting in notification. The HCW has pressure and a professional commitment to actions which maximise positive public health outcomes but is the individual that is least dependent on the exchange for maximising benefits within his, her or their personal relationships. The index client here is described as the most vulnerable, with the most to lose upon notification because not only do they have confirmation of an illness, they are expected to disclose this to partners to prevent onward transmission, which may – for many reasons – be complex and life-changing. Whereas, the notified partner is seen as relatively powerful, and able to influence the maximisation or loss of valued resources depending on how they use, or can use, the information and whether they access services. External, contextual forces such as trust, rewards, relationships, reciprocity and the institutional working environment influence the whole basis for exchange, and previous as well as ongoing experiences with these dimensions are likely to influence the notification process (Figure 1).

## Results

We describe findings from *n* = 15 participants (Table 1) along the 4 dimensions of our conceptual framework (relationships, safety, human rights and professional success) to explain how VAPN for marginalised populations may present barriers to utilising this approach for disclosure. We find that although VAPN is often seen to be effective, notification is also seen as more costly than beneficial within the framework of social exchange. Participants highlighted that the scope of the VAPN policy was not broad or nuanced enough for those who needed differentiated services, such as vulnerable women, MSM, and children. Women were seen as vulnerable to violence following partner notification and lacked access to adequate gender-based violence support. Upon HIV status notification, MSM could face exclusion from important social networks leading to further marginalisation, particularly in places where same-sex relationships are illegal. Age-appropriate VAPN assistance was also considered unavailable for sexually active children, especially those in a sexual relationship with someone significantly older. Participants from the African region spoke more about implementation, such as how VAPN works in clinics and issues relating to confidentiality, whereas the participants from the global north, spoke – in general – about targets, communities and vulnerability.

**Table 1.** Participant characteristics.

Characteristic	Number (%)
Female	5 (25)
Male	10 (75)
Age group (years)	
30-40	3 (20)
41-50	6 (40)
51-60	3 (20)
Not reported	3 (20)
Operational level	
International	5 (33.33)
National	6 (40)
Community	4 (26.67)
Country	
Eswatini	1 (6.67)
Kenya	1 (6.67)
Malawi	3 (20)
Sierra Leone	1 (6.67)
South Africa	1 (6.67)
Switzerland	3 (20)
Tanzania	2 (13.33)
Uganda	1 (6.67)
United Kingdom	1 (6.67)
Zimbabwe	1 (6.67)
Total	15 (100)

### Safety

The physical and emotional safety of women upon notification was a primary concern of most participants. VAPN was seen as an additional risk factor for women in general, but especially those in contexts of high HIV risk and vulnerability. Women were described as particularly at risk of physical violence and lacked the agency to access support services against gender-based violence, where they existed in sub-Saharan Africa. Women were seen as reticent to notify partners, as VAPN could increase their vulnerability in terms of exclusion from the family, homelessness, isolation and longer-term safety.

Women have a risk of violence that is exceptionally high. And gender-based violence services are not routinely available. So, I think it really is a call to action for these countries and not just for the HIV program but in general to say we need to be doing something about gender-based violence. (International level, Female)

Yah, so like this woman has been chased out of her home, so who handles this matter? That process should be in place and I think more security in terms of how are these index clients being protected? How are people who mentioned their partners being protected? It's not about the confidentiality, it's about the protection, the real protection. (Working at both the national and community levels, Male)

Participants also highlighted concerns in relation to children. The emotional vulnerability and the emotional burden of the notification process – from the perspective of the notifier and the notified – was thought to be overwhelming and unsafe for children, some of whom could be as young as 10 years old. The expectation that children would be able to navigate the VAPN process safely was questioned, as it could be that the sexual partner was someone from their school and of a similar age, or it could be an adult, a much older or younger student, and almost always out of the ‘protection’ of a marriage or long-term relationship. Being notified of a possible HIV status as a young child without emotional support was seen as problematic, alongside whether a child could have the agency and knowledge to then reach a clinic, access care and understand the implications of a HIV diagnosis.

… But again, if they are like learners [students] in school, for example secondary school or colleges or even primary school, kids are involved in sex as early as 10 sometimes 12 or 14. So if in the event that they have infected each other [with HIV], how do they now reach out to a fellow learner whom they don't have like long-term relationships like marriage or something. How do they now reach out to them to get them to the facility for the test? I'm seeing because I mean we look at the situation under normal circumstances, if my kid gets infected at school by say a boyfriend or a girlfriend, and then they are being asked to go to the facility for a test and they test [HIV-]positive and we know who infected them, yeah under normal circumstances it would be difficult to grasp that, you understand what I'm saying? (National level, Male)

They [the child] would need to understand who is a contact? Because we are looking at a contact in the sense of being a child, being a sexual partner, all those types of information were being provided so that they should understand, who is a contact of an index client in the context of VAPN by looking at sexual and partner network, we are looking into that type of thing. (Community level, Male)

### Relationships and Social Networks

Notification as a source of loss of relationships and social networks was a major reoccurring theme. Participants discussed this mostly in relation to MSM and – albeit to a lesser extent – children. Participants said that as a public health approach, VAPN was good, but at an individual level it was problematic, especially for MSM that had spent a long time building safe, supportive social networks where they had achieved a sense of belonging. Notification was thought to place clients with the dilemma, that if they notified their sexual partners of their status, they may lose the community they had fought hard to achieve. The fear of loss was particularly relevant in settings where same-sex relationships are illegal. A loss of relationships was described as equivalent to a loss of health which – in their opinion – made the process lose value.

I mean I think that when somebody tests for HIV, that's the constant negotiation, right? So, if you know we like to say “oh it's better to know [about HIV exposure]” but sometimes it might not be better to know. Sometimes it might not be better at that specific time for that person to know. Is it [VAPN] better in terms of a public health outcome? Of course, it is, a 100% it is. There is no mitigation there, but really what is somebody's health if they have no social system? If they lose access to their social and economic sometimes, like life. Then they can’t still sustain themselves and become, be healthy, remain healthy. So, you can get that person on ART [antiretroviral therapy] but you can’t give them back a social system. So really, you know, I think that that level of understanding needs to be taken into account when you are designing programs like VAPN. (Working at both community and international levels, Male)

Participants also explained that VAPN did not function as well where index clients had multiple short-term and or concurrent relationships. In relationships where long-term contact is not maintained, and where phone numbers and place of residence are unknown, locating and contacting partners becomes resource-intensive and beyond the capacity of HCWs. Therefore, participants explained that emphasis is placed heavily on the index client to find their partner and make contact alone. Contact may be further complicated by the index-patient not knowing the partners well enough to discuss something like HIV, and having limited support from the HCW regarding disclosure and testing.

I think one of the reasons why, why South Africa is struggling is because, I think, it's simply by how relationships are organized and how people live or don't live together. We've got a very large population here that either doesn't live together or is in a single sort of relationship status. So, even when you approach someone to say, look who's your partner or mobilize your partner, it's quite difficult for them to do so because, you know, these are not the kind of relationships quite often that that allow one partner to say to the other: “Look let's come and go and test together”. We do try to bring couples in, so, but it's always done through, through the index patient basically. (National level, Male)

This was also relevant for FSWs who would face challenges in locating and notifying their clients. FSWs may not want to reach clients as it may harm their professional reputation, and clients may not provide their real contact details for the fear of being linked to FSWs.

They [FSWs] have more than one client. For those … where their [index] clients are all their contacts or what have you, it would be easy, but it will only prove to be a challenge for those [index clients] who have like short-term relationships maybe for a night and they don't know where, their client is going next day or the other week. It'll be only problematic in such a situation, but where we can we revise to say if you can be taking the contact details of your clients, maybe that would help again, but I'm not sure how many clients would be honest to give true contact details about themselves. (National level, Male)

### Concerns About Rights and Confidentiality

Our participants described many scenarios where the rights of clients – particularly in relation to confidentiality and privacy – were ignored because HCWs wanted to ascertain whether the index client had notified their partner(s), and therefore, contacted partners without consent. While some of this unauthorised contact was linked to the HCWs’ need to meet globally and nationally-set VAPN targets, other examples of contact were because the HCW believed that reaching the client at any cost was best for the clients’ health. Participants explained that VAPN had been introduced in many places with VAPN targets and figures that should be reached, and preferably exceeded. These targets were thought to prompt HCWs into forcing patients into disclosing partner contact details so that the HCWs could assist in notifying partners. In some instances where clients had not given consent for their partners to be called, our participants described clients as devising their own strategies to prevent the HCW from contacting them. Blocking and ignoring calls were the most frequent strategies described.

Like we have had clients who …, if they have told you not call them, and you have pressure for targets, and you already know that this client is positive and most likely if they give me their sexual partners most likely I'll be able to get HIV-positive clients and add up to my target, then there are people who will call people who do not give the consent of being called which is very annoying. So when a client doesn't want to be called, they shouldn't be called. If you do call them, …, you always get reactions. Yah. And they block you. Sometimes we have counsellors who have been blocked by clients because of calling them. (National level, Female)

### Guarding HIV Status

Guarding the HIV status of individuals – whether positive or negative – was seen as essential. Participants said there were many and varying complex workarounds used to support notification, avoid notification, and avoid testing. These actions were common behaviours from all 3 parties: the HCW, the index client, and their partner. Participants said HCWs would pretend that they did not know the client or their status to facilitate couples counselling, protect the index client from being found out to have lied or not told the partner about his or her HIV status.

In some of the examples, we call in a partner and they come both and we pretend as if we do not know the index client. So, it's like they are both coming for the first time to test for HIV. So, because we don’t want to show that it is the partner but it is the index client who gave us your number. That would mean if this woman or this husband has not disclosed would mean we are saying he has been living with HIV and he hasn’t told you. So, we made out some sort of closure and privacy to make sure that we protect our clients. (Community level, Female)

But again, another challenge that we realized later was … the client brings the partner but probably the partner had been taking medication [ARV] or he actually knows his status or her status but he does not want to disclose that he has been taking [ARV] medication then he pretends that not knowing his status and comes and retests. So, we had some circumstances where we enroll someone as a new client at this facility and because we matched the database and we realize he has been taking medication from a different facility somewhere. So basically, because he never wanted to be seen that he knew his status or he has been taking ARVs so he tests again. (Working at both national and community levels, Male)

Participants also said that HCWs asked clients to bring their partners, and clients themselves often brought their partners to the health care facility under the guise of visiting for reasons unrelated to HIV.

So maybe a partner convinces the partner to come in not for HIV testing actually, just to go to the facility for other business. And then there were a few people that knew they were coming for HIV testing but majority did not know they were coming for HIV testing. So, they come to the facility and then they get to meet, because one of the principles was to protect the index client who gave the contact of the partner. Because one thing we realized people were living together and partners did not know each other's [HIV] status. (National level, Male)

Participants also explained that the practice of ‘proxy testing’ was common in many places, where – especially men – would infer that the status of their partner would be the same as their own. Participants said that men avoided testing for HIV and would guard their status until their partner would *have* to test due to pregnancy, illness, and an awareness that their partner was engaging in multiple sexual relationships. Even with a ‘proxy’ result, women with a negative status would still not have an accurate understanding of their partner’s status and remain vulnerable to infection. With this in mind, participants said that when a woman was able to bring her partner into the health care facility, it would be a suitable basis on which to broach testing in a safe environment, and the benefits that knowledge of HIV status could bring. HIV self-testing at home was also identified as a positive support to clinic-based testing methods.

Because culturally, traditionally men in Tanzania use women's testing status as the proxy to their own status. So, when a man says my wife was tested for HIV at antenatal care and was found [HIV-] negative then I am negative, then that's it. So, this is because when a woman gets an opportunity to bring their partner along and they were tested and probably found [HIV-] positive or negative but it was actually an empowering experience when these two partners were brought together and had an opportunity to discuss and test together. So, there are women who feel they have been helped. And in the context where a woman has tried so many times to bring their husband to come for [HIV] testing and for some reason if she has failed, and then this intervention gave her an approach to go and test at home, it was also a positive experience that women were so relieved that, ‘Okay I was worried, I wanted my husband to get tested. Now he is tested and I know his status. So, we count it a positive experience. (Working at both national and community levels, Male)

### Counselling Skills

The ability of HCWs delivering VAPN to counsel appropriately was a major theme within the dataset. While some healthcare providers were described as being target-driven and selected people to counsel using a VAPN approach because of the ease of reaching partners, others did not, and chose to use VAPN because their interpersonal and professional skills were good enough to do so. It seems that those providers and expert clients who belonged to the same group (for example, MSM) or were living with HIV themselves seemed to be better equipped to do this type of counselling.

So, this relationship between the expert clients [HIV positive individuals with much experience with prevention and treatment that are used for communicating information relating to HIV] and this new client it made it easy because they discuss a lot of things, so clients are more likely to disclose [their HIV status] to these expert clients than to someone else. I mean they share a lot, they discuss and this new client feels more open to this because he also, he is living with HIV and now, I am living with HIV, so, some sort of we share something in common. So, at some point these community people were used effectively, and they managed to bring people and open [up]. (Working at both national and community levels, Male)

Good counselling skills were considered to be of particular importance with MSM and children, as these groups require specialist communication approaches in often intimate settings, such as the home, and need individuals with whom the client can relate to. Understanding the broader context of the clients’ life, any other illnesses they may have and their individual needs should be integrated into VAPN, but this information was not always available or used.

… because of that [HIV] stigma between them [counsellor and the client], and the [VAPN] program in Kenya, we use peer educators. So, for instance, if you are MSM, we have to use a peer in MSM to get you. So those are the challenges that when a counsellor calls an MSM who is living with HIV and most probably they already know the peer, they will think that the peer will have information and will know about their HIV-status. And they do not want to bring them to be tested. (National level, Female)

Participants said that the broader family context of an HIV-positive child who needed support with notification should be understood. Building a picture of their home environment was important for HCWs, especially in relation to who it was that needed to be notified, and how notification would be managed alongside supporting a child and relationships within the household.

I think we should elaborate a little bit on the family approach. I think it's quite important because it also takes children into account, and it looks at an individual more holistically. Even if the partner is staying somewhere else or they don't have a stable partner, it's something you, you want to know along with the relationship, relationship status or type and, the HIV-status within that family or within that household. I think we don't often take that into account. And it would be incredibly helpful, if that were done a bit more. So, you draw basically, draw the structure of the households and then look at who is tested who is positive both for TB and HIV. (National level, Male)

## Discussion

We present findings from 15 medical and health professionals who have worked or are working in the VAPN field across the community, national and international levels. We use the theory of social exchange to highlight the resources people value within the context of VAPN and how experiences can influence the level of risk someone is prepared to experience before they enter or leave the exchange process. We highlight that safety, relationships, health rights, HIV status, and counselling skills to motivate clients to engage in VAPN hold value within the context of disclosure, and should be considered within a more holistic and context-specific approach with the index client, their partner and the HCW.

Our findings resonate with evidence where safety is a primary concern of and for women, children, and MSM in the HIV care continuum.^31–33^ The broad context of the testing environment reveals vulnerabilities in the form of gender-based violence,^34,35^ stigma,^36^ arrest and exclusion,^37,38^ which are exposed when trying to engage with VAPN services and specifically with notification. More research is required to ascertain how VAPN specifically dissolves relationships or undermines support in a context where numerous factors in relation to HIV infection could increase the risk of harm and exacerbate vulnerabilities.

We see similarities in our findings with that of Vermandere et el. (2021), where complex, multiple and non-traditional type relationships – which may be more common in certain marginalised populations – can prevent VAPN functioning because partners cannot be found, there are multiple partners and access is restricted by partners.^39^ However, there are examples of where VAPN has been a successful approach in marginalised groups not mentioned by our participants, such as refugees^40^ and incarcerated men.^41^ Following approaches such as Impart APN, where an APN-trained nurse and community outreach healthcare worker ‘locate and notify their partners without revealing the identity of who had named them’ (page 3, 2023) and then contact named partners with important health information that should be discussed in person,^41^ could be a more appropriate approach.

We observe that the value placed on maintaining relationships – particularly for some women, MSM, and children – is perceived as exceeding the personal or public health need and expectation to test and notify, as this may undermine relationships within family units^42^ and, for example, in MSM communities because of stigma or criminalisation of same-sex relationships.^39^ This remains a constant factor in understanding the best approaches for HIV prevention and care, and the overall well-being of marginalised groups.^43^ This is comparable with children and women dependent on caregivers and family relationships for their well-being, and men who fear the loss of employment^44^ or their female partners, as the action of notification may compromise the relationship and pose greater or equivalent health risks.^45^ However a child may perceive the loss of resources upon notification as greater than an adult, but their health outcomes can be better once HIV status is known because of early access to treatment and age-appropriate support services.^46^

The well-being and health of individuals derived from being part of a community is long established and understood within the social determinants of health, where the systems shaping daily life influence how health is experienced.^47^ As with work from Hatzenbuehler and Pachankis (2016), we observe that in relationships where one or more party is marginalised or particularly vulnerable – such as a child – these systems can exacerbate marginalisation and offer limited protection where it is required.^48^ Our work highlights how the VAPN process – as described by our participants – does not (via policy and practice guidance) adjust for those who are vulnerable, and where individual and alternative approaches may be required. The provision of good quality, culturally specific sex education^49^ alongside family or household approaches, as demonstrated by Simon et al (1999)^42^ may be one method, where HCWs have specialised training to engage and support diagnosis and notification, and advise, support and initiate child protection procedures (if available) where a young child may be in a sexual relationship.^46^

Interviews suggested that violations of confidentiality and other patient rights may be common in SSA settings where VAPN is implemented, and that this is closely related but runs in conflict with the value placed on HIV status and professional success. There is a constant interchange of approaches to protect the HIV status of people as well as to support notification, which reflects the nuance in an individuals’ care needs as well as a unique understanding of the community. While tailored support via, for example, peer educators and expert clients is commonplace, the nuance required in care provision for marginalised groups is a largely uncaptured dynamic, but has been highlighted in other studies where ‘tinkering’ with the specified procedures is one way to provide a more unique, client-centered approach.^50^ Tinkering refers to undocumented, small, patient-specific and changeable adaptation to programs. These changes are frequently missed and excluded as viable changes that can be made to improve implementation and patient outcomes.^50^

While protection of clients and their HIV status is evident throughout our data, it is also clear that HCWs, as observed in other studies such as the opt-out strategy for HIV testing in pregnancy,^51^ can be driven to push the boundaries of health rights to achieve external targets, contradicting client-centered approaches.^52^ Emphasis on differentiated care or ‘tinkering’ within the country-specific guidelines may prevent violations of health rights by allowing the space required for interpretation of needs and wishes in relation to VAPN that could lead to higher uptake of VAPN services and help achieve the required targets.

Our interpretation of the social exchange framework is useful for highlighting the exchange regarding the maximisation or loss of valued resources between the index partner, the partner and the HCW that is assisting in notification. Trust, rewards, relationships and reciprocity influence the whole basis for exchange, and previous and ongoing experiences with these dimensions are likely to influence the notification process.^29^ VAPN policy appears to be based on an individual's ability to make decisions and rational choices but the framework shows how this decision-making process exists within a social world where the ability to make a rational choice has often been removed. This removal is not only at the level of the HCW but also by other parties in the relationship, and then by the complex interplay between trust, rewards, relationships, the institutional working environment and reciprocity. VAPN assumes – for public health reasons – that notification will result in the reward of a negative HIV status or direction to ART and HIV care for an HIV-positive status, and that individuals can trust the HCWs and predict the response of partners upon notification. But for marginalised groups, we see that the previous and ongoing experiences with these dimensions have created a context where people cannot trust, have limited experience of positive reciprocity and fear that the risks associated with safety and relationship loss, far outweigh that of any reward. The HIV status – be it positive or negative – is something of value because of its perceived influence on other valued resources. Therefore, when entering the exchange process within VAPN, it has to be understood what the personal and public outing of HIV status means for the index client and the notified, so that the process can be adapted accordingly to maintain the value people allocate to certain resources. Integrating human rights principles with prevention and care options such as pre-exposure prophylaxis (PrEP) may help with reciprocity and reward, as well as acknowledging valued resources will change over the life course alongside prevention, treatment and notification needs. Ensuring that HCWs place value on maintaining the confidentiality of marginalised clients is essential as this impacts their long-term safety and ability to live a healthy life. Recognising that children with limited previous experience may struggle with assessing trust, rewards, relationships, the professionalism of HCWs, reciprocity, and their reliance on adult care should be integral to VAPN support for children.

## Limitations

Our study has a small sample of actors within the VAPN field. We emphasise that the research is based on interviews with experts and key players who may approach VAPN with differing levels of experience and commitment. As such, the results speak only to the perceptions held by this group. We conducted online interviews at the beginning of the COVID-19 pandemic, and many of the health professionals contacted were busy with the initial COVID-19 response and unable to participate in our interviews. As this is a qualitative study, we cannot show any kind of frequency or power in relation to how trust, rewards, relationships and reciprocity influence the VAPN process, but feel the qualitative description and interpretation highlight the importance of these dimensions when considering VAPN for marginalised and vulnerable populations in the future.

## Conclusion

VAPN can be a useful tool for HIV but marginalised communities have complex care needs which the current policy does not support. Embedding understandings of identity, belonging within relationships, and safety into VAPN could address individual priorities and needs. Clear policy, community support networks, tailored care for children, and family-orientated approaches to HIV notification may overcome issues relating to vulnerability and marginalisation.

## Supplemental Material

sj-docx-1-jia-10.1177_23259582241272059 - Supplemental material for ‘You Can Get That Person on ART but You Can’t Give Them Back Their Social System’: A Qualitative Analysis of Voluntary Assisted Partner Notification for HIV for Marginalised and Vulnerable Populations Supplemental material, sj-docx-1-jia-10.1177_23259582241272059 for ‘You Can Get That Person on ART but You Can’t Give Them Back Their Social System’: A Qualitative Analysis of Voluntary Assisted Partner Notification for HIV for Marginalised and Vulnerable Populations by Kate Bärnighausen, Astrid Berner-Rodoreda, Maureen McGowan, Mark Donald Reñosa, Caroline Mtaita and Florian Neuhann in Journal of the International Association of Providers of AIDS Care (JIAPAC)

sj-docx-2-jia-10.1177_23259582241272059 - Supplemental material for ‘You Can Get That Person on ART but You Can’t Give Them Back Their Social System’: A Qualitative Analysis of Voluntary Assisted Partner Notification for HIV for Marginalised and Vulnerable Populations Supplemental material, sj-docx-2-jia-10.1177_23259582241272059 for ‘You Can Get That Person on ART but You Can’t Give Them Back Their Social System’: A Qualitative Analysis of Voluntary Assisted Partner Notification for HIV for Marginalised and Vulnerable Populations by Kate Bärnighausen, Astrid Berner-Rodoreda, Maureen McGowan, Mark Donald Reñosa, Caroline Mtaita and Florian Neuhann in Journal of the International Association of Providers of AIDS Care (JIAPAC)

sj-docx-3-jia-10.1177_23259582241272059 - Supplemental material for ‘You Can Get That Person on ART but You Can’t Give Them Back Their Social System’: A Qualitative Analysis of Voluntary Assisted Partner Notification for HIV for Marginalised and Vulnerable Populations Supplemental material, sj-docx-3-jia-10.1177_23259582241272059 for ‘You Can Get That Person on ART but You Can’t Give Them Back Their Social System’: A Qualitative Analysis of Voluntary Assisted Partner Notification for HIV for Marginalised and Vulnerable Populations by Kate Bärnighausen, Astrid Berner-Rodoreda, Maureen McGowan, Mark Donald Reñosa, Caroline Mtaita and Florian Neuhann in Journal of the International Association of Providers of AIDS Care (JIAPAC)

sj-docx-4-jia-10.1177_23259582241272059 - Supplemental material for ‘You Can Get That Person on ART but You Can’t Give Them Back Their Social System’: A Qualitative Analysis of Voluntary Assisted Partner Notification for HIV for Marginalised and Vulnerable Populations Supplemental material, sj-docx-4-jia-10.1177_23259582241272059 for ‘You Can Get That Person on ART but You Can’t Give Them Back Their Social System’: A Qualitative Analysis of Voluntary Assisted Partner Notification for HIV for Marginalised and Vulnerable Populations by Kate Bärnighausen, Astrid Berner-Rodoreda, Maureen McGowan, Mark Donald Reñosa, Caroline Mtaita and Florian Neuhann in Journal of the International Association of Providers of AIDS Care (JIAPAC)
