# Supplementary material for: ‘You Can Get That Person on ART but You Can’t Give Them Back Their Social System’: A Qualitative Analysis of Voluntary Assisted Partner Notification for HIV for Marginalised and Vulnerable Populations
Source: J Int Assoc Provid AIDS Care. 2024 Sep 9;23:23259582241272059. doi: 10.1177/23259582241272059 (PMC11403698; doi:10.1177/23259582241272059)
Supplement: sj-docx-2-jia-10.1177_23259582241272059 - Supplemental material for ‘You Can Get That Person on ART but You Can’t Give Them Back Their Social System’: A Qualitative Analysis of Voluntary Assisted Partner Notification for HIV for Marginalised and Vulnerable Populations [file sj-docx-2-jia-10.1177_23259582241272059.docx]

In-depth Interview guide

Community level stakeholders / providers

As we described in the information sheet, everything you share will be kept confidential and your names will not be recorded. Just as a reminder our discussion will probably last around 60 minutes. Some of the questions I will ask you may not want to answer and that is fine. Remember that your participation is completely voluntary. Also please keep in mind that there are no right or wrong answers, I am interested in anything you can share with me. Do you have any questions before we begin? May I start the recording? *[Start recording]*

**Good [afternoon/morning] thank you for participating today**!

The purpose of this project is to develop a better understanding of voluntary assisted partner notification from the perspective of stakeholders, policy makers and implementers working in countries where VAPN is being implemented.

I have asked you to meet with me in the hopes of learning more about your personal opinions and experiences with VAPN in terms of the barriers and facilitators to implementation, perceptions around rights and disclosure and opportunities for improvement.

We will be taking notes and also recording our conversation so that we can accurately capture and report your views. Your comments will be combined with those from other interviews.

1. Please tell me a little bit about yourself. What is your current role?
2. For how long have you worked in the field of HIV?
3. How was VAPN introduced at community level?
4. How would you describe the process of introducing VAPN as part of the HIV testing strategy?
5. Did you receive training on how to communicate VAPN?
6. How were the communities informed about the implementation of VAPN?
7. How were national organizations (i.e. National Association for People Living with HIV and AIDS) informed about the implementation of VAPN?
8. What are your thoughts about this process?
9. How do you report VAPN implementation findings to the country level?
10. What costs occur at implementation level for VAPN? (optional, depending on interviewee)
11. What do you believe facilitates the implementation of VAPN?
12. What do you believe hinders the implementation of VAPN?
13. What do you believe the outcomes of VAPN have been?
14. Have you recognized any positive effects?
15. How do you measure these effects?
16. Have you recognized any adverse effects?
17. How do you measure these effects?
18. What yield have you noticed, if any?
19. What do you think the uptake of VAPN has been in those you have recommended it to?
20. Could you take me through the process of VAPN?
21. How do you ask clients to provide contact information for sexual partners?
22. Please walk me through an example where a client has had a positive experience with VAPN.
23. PROBE: What made it positive?
24. PROBE: Why do you remember this particular example?
25. Please walk me through an example of where a client had a negative experience with VAPN.
26. PROBE: What made it negative?
27. PROBE: Why do you remember this particular example?
28. How do you view VAPN from a human-rights perspective?
29. What guidelines do you follow to maintain the voluntary nature of VAPN?
30. What guidelines do you follow to maintain confidentiality and disclosure?
31. What guidelines do you follow to prevent or address adverse effects (i.e. IPV)?
32. How does actual implementation compare with the guidelines?
33. Where do you see opportunities for improvement of VAPN?
34. PROBE: Policy level

b) PROBE: Implementation / Clinic level

1. PROBE: Experiences of clients
2. PROBE: Experience of partners of index-client
3. Can you tell me about any other methods of partner notification which you believe to be preferable to VAPN and why?
4. Please tell me about any other thoughts you have regarding VAPN.
5. Is there anything else you would like to add? Any questions that I should have asked you?
