# Supplementary material for: ‘You Can Get That Person on ART but You Can’t Give Them Back Their Social System’: A Qualitative Analysis of Voluntary Assisted Partner Notification for HIV for Marginalised and Vulnerable Populations
Source: J Int Assoc Provid AIDS Care. 2024 Sep 9;23:23259582241272059. doi: 10.1177/23259582241272059 (PMC11403698; doi:10.1177/23259582241272059)
Supplement: sj-docx-3-jia-10.1177_23259582241272059 - Supplemental material for ‘You Can Get That Person on ART but You Can’t Give Them Back Their Social System’: A Qualitative Analysis of Voluntary Assisted Partner Notification for HIV for Marginalised and Vulnerable Populations [file sj-docx-3-jia-10.1177_23259582241272059.docx]

In-depth Interview guide - Country level stakeholders

As we described in the information sheet, everything you share will be kept confidential and your names will not be recorded. Just as a reminder our discussion will probably last around 60 minutes. Some of the questions I will ask you may not want to answer and that is fine. Remember that your participation is completely voluntary. Also please keep in mind that there are no right or wrong answers, I am interested in anything you can share with me. Do you have any questions before we begin? May I start the recording? *[Start recording]*

**Good [afternoon/morning] thank you for participating today**!

The purpose of this project is to develop a better understanding of voluntary assisted partner notification from the perspective of stakeholders, policy makers and implementers working in countries where VAPN is being implemented.

I have asked you to meet with me in the hopes of learning more about your personal opinions and experiences with VAPN in terms of the barriers and facilitators to implementation, perceptions around rights and disclosure and opportunities for improvement.

We will be taking notes and also recording our conversation so that we can accurately capture and report your views. Your comments will be combined with those from other interviews.

1. Please tell me a little bit about yourself. What is your current role?
2. For how long have you worked in the field of HIV?
3. When did your country start to get involved with VAPN?
4. Can you take me through the process of introducing VAPN as a national strategy?

PROBE: How have you translated global VAPN goals to a national policy?

PROBE: How have you translated the national VAPN policy to the community level?

PROBE: How was VAPN introduced at the community level?

(national simultaneous introduction, stepwise /phased introduction)?)

1. How important is VAPN in the overall HIV testing strategy?
2. What is the national coverage of VAPN?

PROBE: How many provinces/counties are covered?

PROBE: Who are the main implementers (NGOs, Health facilities…)

1. Please tell me about any incentives there are for those centers implementing the VAPN recommendation? (probe in terms of manpower, time and cost)
2. What is the estimated cost of implementing VAPN?
3. Do you have reporting guidelines for VAPN?

PROBE: How do those implementing VAPN at the community level report VAPN to you?

PROBE: How do you report country-level findings to global actors (i.e. WHO, CDC)

1. How is VAPN conducted in communities with small populations?
2. What do you see as facilitators to implementation of the VAPN recommendation?
3. What do you see as barriers to the implementation of the VAPN recommendation?
4. What do you believe that outcomes of VAPN are?
5. Have you recognized any positive effects?
6. How do you measure these effects?
7. Have you recognized any adverse effects?
8. How do you measure these effects?
9. Can you say anything about the yield of VAPN so far?
10. How do you view VAPN through a human-rights point of view?
11. What is the programmatic guidance for maintaining the voluntary nature of VAPN?
12. What is the programmatic guidance for maintaining confidentiality and unintended disclosure?
13. What are the guidelines for preventing and addressing adverse effects (i.e. Intimate Partner Violence)?
14. How does implementation compare with the guidelines?
15. Please describe any incidences where you have noticed or heard of adverse human-rights effects in the context of VAPN
16. Where do you see opportunities for improvement of VAPN?
17. PROBE: Policy level
18. PROBE: Implementation / Clinic level
19. PROBE: Experiences of clients
20. PROBE: Experience of partners of index-client
21. Can you tell me about any other methods of partner notification which you believe to be preferable to VAPN and why?
22. Please tell me about any other thoughts you have regarding VAPN.
23. Is there anything else you would like to add? Any questions that I should have asked you?
