# Supplementary material for: ‘You Can Get That Person on ART but You Can’t Give Them Back Their Social System’: A Qualitative Analysis of Voluntary Assisted Partner Notification for HIV for Marginalised and Vulnerable Populations
Source: J Int Assoc Provid AIDS Care. 2024 Sep 9;23:23259582241272059. doi: 10.1177/23259582241272059 (PMC11403698; doi:10.1177/23259582241272059)
Supplement: sj-docx-4-jia-10.1177_23259582241272059 - Supplemental material for ‘You Can Get That Person on ART but You Can’t Give Them Back Their Social System’: A Qualitative Analysis of Voluntary Assisted Partner Notification for HIV for Marginalised and Vulnerable Populations [file sj-docx-4-jia-10.1177_23259582241272059.docx]

In-depth Interview guide - Global stakeholders and policy makers

As we described in the information sheet, everything you share will be kept confidential and your names will not be recorded. Just as a reminder our discussion will probably last around 60 minutes. Some of the questions I will ask you may not want to answer and that is fine. Remember that your participation is completely voluntary. Also please keep in mind that there are no right or wrong answers, I am interested in anything you can share with me. Do you have any questions before we begin? May I start the recording? *[Start recording]*

**Good [afternoon/morning] thank you for participating today**!

The purpose of this project is to develop a better understanding of voluntary assisted partner notification from the perspective of stakeholders, policy makers and implementers working in countries where VAPN is being implemented.

I have asked you to meet with me in the hopes of learning more about your personal opinions and experiences with VAPN in terms of the barriers and facilitators to implementation, perceptions around rights and disclosure and opportunities for improvement.

We will be taking notes and also recording our conversation so that we can accurately capture and report your views. Your comments will be combined with those from other interviews.

1. Please tell me a little bit about yourself. What is your current role?
2. For how long have you worked in the field of HIV?
3. Can you tell me something about the global implementation of VAPN?
4. Can you tell me something about the implementation in sub-Saharan African countries?
5. PROBE: What incentives are provided to countries for implementing the VAPN recommendations?

(Incentives to train additional staff, cost/time needed to locate partners etc.)

1. Please tell me about the information available for VAPN.
2. PROBE: What information is available for implementers?
3. PROBE: What information is available for clients?
4. PROBE: What information is available to the general public?
5. PROBE: Where do you see information gaps?
6. How do you monitor implementation of the 2016 WHO VAPN recommendation?
7. Please tell me about the reporting guidelines for VAPN implementation.
8. What would make VAPN cost-effective in your opinion?
9. PROBE: Which factors need to be considered for cost-effectiveness estimates)
10. Which factors have facilitated VAPN implementation in sub-Saharan Africa in your opinion?
11. Which factors do you think have proved challenging for VAPN implementation in sub-Saharan Africa?
12. What is currently known about the outcomes of VAPN?
13. PROBE: What outcomes have you seen in terms of the HIV epidemic?
14. PROBE: Please tell me about any positive outcomes you can think/know of
15. PROBE: Please tell me about any negative outcomes you can think/know of
16. PROBE: How are these outcomes measured?
17. PROBE: Has VAPN had any effect on yield (identifying more HIV-positive people)?
18. What do you think are the human-rights considerations of VAPN?
19. PROBE: What is the programmatic guidance for maintaining the voluntary nature of VAPN?
20. PROBE: What is the programmatic guidance for maintaining confidentiality and unintended disclosure?
21. PROBE: What are the guidelines for preventing and addressing adverse effects (i.e. Intimate Partner Violence)?
22. PROBE: How do you think implementation compares to the guidelines?
23. What adverse human-rights effects have you been made aware of in the context of VAPN?
24. Do you think any changes are needed in implementing VAPN? (if yes, probe)
25. PROBE: What do you think can be done to enhance the experience of clients guided to VAPN?
26. PROBE: What could enhance the experience of partners of index-clients?
27. Can you tell me about any other methods of partner notification which you believe to be preferable to VAPN and why?
28. Please tell me about any other thoughts you have regarding VAPN.
29. Is there anything else you would like to add? Any questions that I should have asked you?
